# Impact of temperature and state-of-charge on long-term storage degradation in lithium-ion batteries: an integrated P2D-based degradation analysis

**DOI:** 10.1039/d5ra03735b

**Published:** 2025-07-02

**Authors:** Mohammed Asiri, Munthar Kedhim, Vicky Jain, Suhas Ballal, Abhayveer Singh, V. Kavitha, Nargiza Kamolova, Milad Nourizadeh

**Affiliations:** a Department of Clinical Laboratory Sciences, College of Applied Medical Sciences, King Khalid University Abha Saudi Arabia; b College of Pharmacy, The Islamic University Najaf Iraq muntherabosoda@iunajaf.edu.iq; c Marwadi University Research Center, Department of Chemistry, Faculty of Science, Marwadi University Rajkot-360003 Gujarat India; d Department of Chemistry and Biochemistry, School of Sciences, JAIN (Deemed to be University) Bangalore Karnataka India; e Centre for Research Impact & Outcome, Chitkara University Institute of Engineering and Technology, Chitkara University Rajpura Punjab 140401 India; f Department of Chemistry, Sathyabama Institute of Science and Technology Chennai Tamil Nadu India; g Department of Chemistry and Its Teaching Methods, Tashkent State Pedagogical University Tashkent Uzbekistan; h Young Researchers and Elite Club, Ilam University Ilam Iran miladnourizadehacademic@gmail.com; i Department of Medical Analysis, Medical Laboratory Technique College, The Islamic University of Al Diwaniyah Al Diwaniyah Iraq; j Department of Medical Analysis, Medical Laboratory Technique College, The Islamic University of Babylon Babylon Iraq

## Abstract

This study utilizes a Pseudo-Two-Dimensional (P2D) model to predict calendar aging in LiFePO_4_/graphite lithium-ion batteries, emphasizing temperature and state-of-charge (SOC) impacts. Implemented in COMSOL Multiphysics, the P2D framework simulates solid electrolyte interphase (SEI) growth and electrolyte conductivity loss, driven by parasitic redox reactions at the electrode–electrolyte interface, modeled using Arrhenius and Tafel kinetics. Validated against experimental data across five temperature–SOC conditions, the P2D model achieves root mean square errors below 0.9. Results show synergistic degradation, with SEI thickness exceeding 300 nm and conductivity loss over 20% after 36 months at 55 °C and 90% SOC. Higher SOCs intensify SEI growth due to electrolyte instability at elevated anode potentials. This P2D-based, chemically grounded approach provides mechanistic insights into storage degradation, enabling optimized battery management and storage strategies to enhance lifespan and reliability for electric vehicles and grid applications.

## Abstract 2

A P2D-based model reveals how temperature and SOC drive SEI growth and electrolyte degradation in LiFePO_4_/graphite pouch cells, supporting predictive storage strategies to reduce long-term capacity fade.

## Graphical abstract

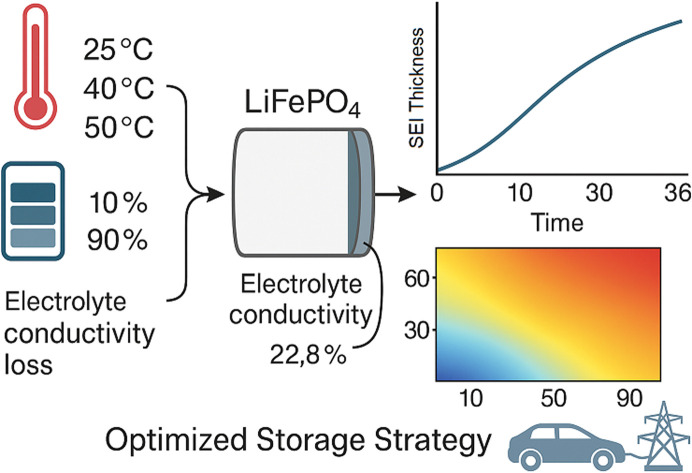

## Introduction

Lithium-ion batteries (LIBs) have become the dominant energy storage technology in portable electronics, electric vehicles (EVs), and grid-scale applications due to their high energy density, long cycle life, and relatively low self-discharge rate.^1–3^ However, their performance inevitably deteriorates over time, even when not in use (a phenomenon known as calendar aging). Calendar aging is particularly critical for applications such as EVs, where batteries often remain idle for extended periods under various temperatures and state-of-charge (SOC) conditions.^4–7^

Aging mechanisms in lithium-ion batteries (LIBs) can be broadly classified into two fundamental categories: cycle aging and calendar aging. Cycle aging arises from repetitive electrochemical cycling (*i.e.*, charge and discharge processes) which impose mechanical, thermal, and chemical stresses on the electrodes, leading to degradation phenomena such as active material loss, structural collapse, particle cracking, and lithium inventory depletion due to repeated intercalation/deintercalation cycles.^8–10^ This aging mode is primarily driven by electrochemical, mechanical, and thermal stresses exerted on the electrode materials during the intercalation and deintercalation of lithium ions. Over time, these stresses lead to phenomena such as active material loss, microstructural damage, SEI instability, and lithium inventory depletion, ultimately resulting in capacity fade and increased internal resistance.^9–11^ Recent studies have highlighted that the rate and nature of cycle-induced degradation are strongly dependent on operational parameters such as depth of discharge (DoD), current rate (C-rate), temperature, and state-of-charge (SOC) window.^8,12^ Therefore, accurate modeling and characterization of cycle aging are essential for predicting battery lifetime and guiding the development of optimized cycling protocols that mitigate long-term degradation.^13,14^

In contrast, calendar aging refers to the gradual degradation of battery performance over time when the cell is stored at a constant state-of-charge (SOC), with no external current flow. This aging mode is driven primarily by parasitic side reactions occurring at the electrode/electrolyte interfaces, particularly at the anode side, even under open-circuit conditions. These reactions are accelerated by elevated temperatures and high SOC levels and contribute to the irreversible loss of lithium, resulting in capacity fade and increased internal resistance over time.^6,15,16^

The dominant degradation mechanisms involved in calendar aging include:

• Growth of the solid electrolyte interphase (SEI) on the anode surface due to continuous electrolyte reduction reactions. This process consumes cyclable lithium and increases ionic/electronic resistance.

• Electrolyte decomposition, which leads to the formation of gas and insoluble byproducts, reducing ionic conductivity.

• Passive layer formation and structural degradation of active materials due to slow but persistent reactions with electrolyte solvents or dissolved transition metals.^9,11,12,17,18^

Among these, SEI layer growth is recognized as the most significant contributor to calendar aging in graphite-based anodes.^19^ While the SEI initially forms during the first few cycles to stabilize the anode/electrolyte interface, its continued growth during storage consumes lithium ions and thickens the interfacial resistance layer.^20,21^ This process follows diffusion-limited, sub-linear time dependence, often modeled with a *t*^0.5^ or *t*^0.75^ behavior, and is strongly influenced by temperature and SOC. The exponent 0.5 typically represents purely diffusion-controlled SEI growth, where lithium-ion transport through the SEI layer limits the reaction rate, as described by Fickian diffusion. The exponent 0.75 reflects a mixed diffusion–reaction regime, where both diffusion and interfacial reaction kinetics contribute, often observed in long-term storage due to SEI densification and porosity changes.^22,23^ In this study, the exponent 0.75 was chosen to align with experimental observations of sub-linear SEI growth in LiFePO_4_/graphite cells under prolonged storage, as validated against data from Sui *et al.*^24^

In addition to these insights, long-term empirical studies have provided valuable data on how cell chemistry and electrode composition influence calendar aging. Frie *et al.*^25^ conducted a comprehensive five-year investigation into the calendar aging behavior of nickel-rich NCA 18650 cells equipped with silicon/graphite (Si/C) composite anodes. The study revealed that elevated storage voltages significantly accelerated degradation, primarily due to intensified solid electrolyte interphase (SEI) growth on the high-surface-area silicon particles. This resulted in a marked increase in capacity fade compared to graphite-only systems, underscoring the vulnerability of silicon-rich anodes to interfacial instability during prolonged storage. Similarly, Wang *et al.*^26^ examined the calendar aging of lithium titanate (LTO)-based cells (renowned for their structural stability) under high-temperature storage conditions (60 °C) at different SOC levels. The findings indicated that even in LTO systems, higher SOCs (particularly 100%) led to greater capacity loss and impedance growth, driven by enhanced side reactions and SEI formation at the electrode/electrolyte interface.

Therefore, accurate modeling of calendar aging (especially SEI dynamics and electrolyte conductivity degradation) is crucial for battery lifetime prediction, health estimation, and thermal/storage management in modern battery applications.

Understanding the influence of storage conditions, such as temperature and duration, on calendar aging is essential for predicting battery lifetime and optimizing battery management strategies. Elevated temperatures significantly accelerate degradation reactions, which can lead to rapid performance deterioration if not properly managed.^27^ Experimental investigations have shown that even moderate increases in storage temperature can markedly reduce battery lifespan.^28^

Physics-based modeling frameworks (particularly pseudo-two-dimensional (P2D) models) have demonstrated significant efficacy in capturing the intricate interplay among electrochemical, thermal, and aging phenomena in lithium-ion batteries (LIBs).^14,29^ In contrast to empirical or data-driven approaches, P2D models are grounded in fundamental transport and reaction equations, allowing for the resolution of spatial and temporal distributions of critical internal state variables. These include lithium-ion concentration gradients within electrodes and electrolyte phases, local overpotentials, and temperature fields across the cell architecture. This level of detail enables more accurate and predictive assessments of performance degradation, especially under diverse storage and operational scenarios.^30^ When augmented with sub-models that represent aging mechanisms (such as solid electrolyte interphase (SEI) layer growth, lithium plating, and active material loss) P2D frameworks become powerful tools for simulating long-term calendar aging behavior. They provide deeper mechanistic insight into capacity fade and resistance growth, which are influenced by electrochemical and thermal inhomogeneities at the micro- and macro-scale.^8^ Consequently, these models serve not only as diagnostic platforms but also as predictive engines for optimizing cell design, storage protocols, and battery management strategies aimed at extending LIB lifespan. Recent advancements in P2D modeling have significantly enhanced the ability to predict lithium-ion battery aging under diverse conditions. For instance, Wickramanayake *et al.*^31^ developed a novel solver for a P2D electrochemical-thermal aging model, incorporating kinetic- and diffusion-limited SEI growth models to estimate capacity fade in real-time with less than 1% error compared to commercial solvers, validated across multiple operational scenarios including 1C discharge/charge cycles. Similarly, Di Prima *et al.*^32^ introduced a pseudo-four-dimensional (P4D) model for cylindrical NMC cells, extending the P2D framework to account for 3D electrochemical and thermal non-uniformities, revealing the impact of SEI growth and cathode electrolyte interface (CEI) formation on calendar aging at 60 °C. Additionally, Su *et al.*^33^ employed a P2D-based electrochemical-thermal coupling model to study capacity fade in M1254S2 button-type lithium-ion batteries, demonstrating that larger anode particle radii lead to thicker SEI films and increased resistance, exacerbating capacity loss over 3000 cycles. These studies demonstrate the robustness of P2D and extended models in capturing electrochemical and thermal dynamics, providing a strong foundation for analyzing calendar aging under varied storage scenarios.

This study aims to implement a physics-based simulation framework using the Pseudo-Two-Dimensional (P2D) model to investigate calendar aging phenomena in commercial LiFePO_4_/graphite cells under various temperature and SOC conditions. By incorporating SEI layer growth and electrolyte conductivity loss into the model, and validating it against long-term experimental data, the proposed approach enables a mechanistic understanding of degradation trends and provides predictive insights for optimizing storage strategies and battery management systems.

## Materials and methods

### Experimental setup

To validate the simulation model of calendar aging in lithium-ion batteries, experimental data were obtained from long-term storage tests reported in Sui *et al.*^24^ These experiments were conducted on cylindrical LiFePO_4_/C cells (nominal capacity: 2.5 Ah), which employed graphite as the negative electrode and LiFePO_4_ as the positive electrode. A total of 15 battery cells were tested under five distinct storage conditions, each combining a specific temperature and state of charge (SOC). The selected stress factors included temperatures of 40 °C, 47.5 °C, and 55 °C, and SOC levels of 10%, 50%, and 90%, enabling investigation of their combined influence on long-term degradation behavior.

To maintain stable environmental conditions throughout the aging process, all cells were stored in precision climate chambers (Weiss WT3-180/40), capable of regulating the ambient temperature within a tolerance of ±0.5 °C. Each group of three cells was subjected to one of the five predefined storage conditions (Table 1). The storage tests extended over duration of up to 43 months for Cases 1–3, and 27 months for Cases 4–5, ensuring sufficient time to observe capacity fade well beyond the conventional end-of-life (EOL) threshold of 20%.

**Table 1:** Storage conditions applied for calendar aging tests of LiFePO_4_/graphite cells^24^

Case	Temperature	SOC level
Case 1	55 °C	50%
Case 2	47.5 °C	50%
Case 3	40 °C	50%
Case 4	55 °C	10%
Case 5	55 °C	90%

To quantify the degradation in capacity, reference measurements were performed at regular intervals of 30 days. Prior to each measurement, the cells were equilibrated to room temperature (25 °C). Discharge tests were conducted following the protocol described in Sui *et al.*,^24^ where cells were discharged at a constant current of 2.5 A (1C rate) to 2.0 V, charged at 1C to 3.6 V, and discharged again at 1C to 2.0 V. The capacity from the final discharge step was recorded as the remaining capacity for retention calculations. While a lower rate (*e.g.*, C/3) with constant-current constant-voltage (CC–CV) charging is often preferred for precise capacity measurements in LiFePO_4_/graphite cells, the 1C-rate protocol was adopted to align with the experimental conditions of Sui *et al.*,^24^ ensuring consistency for model validation.

### Simulation model

The calendar aging behavior of lithium-ion batteries was simulated using a physics-based Pseudo-Two-Dimensional (P2D) electrochemical model implemented in COMSOL Multiphysics (version 6.2). This model captures both macroscopic and microscopic transport processes within the battery and is well-suited for simulating the degradation mechanisms associated with long-term storage. The P2D framework resolves lithium-ion and electronic transport across a one-dimensional spatial domain comprising the graphite anode, porous separator, and LiFePO_4_ cathode, while also modeling intra-particle lithium diffusion in a radial coordinate.

The geometric configuration used in the simulation represents a typical cylindrical 2.5 Ah LiFePO_4_/C cell, consistent with the experimental setup described in ref. 24. The electrode stack included a 50 μm thick graphite anode, a 25 μm separator, and a 70 μm LiFePO_4_ cathode. Relevant material properties were taken from the same reference and supporting literature sources (Table 2).

**Table 2:** Material properties of cell

Initial ionic conductivity	*κ*_0_ = 10 mS cm^−1^
Particle radius (anode)	*r*_p_ = 5 μm
Diffusion coefficient (anode)	*D*_s_ = 3.9 × 10^−14^ m^2^ s^−1^
Particle radius (cathode)	*r*_p_ = 0.1 μm
Diffusion coefficient (cathode)	*D*_s_ = 1.0 × 10^−15^ m^2^ s^−1^
Separator porosity	*ε* = 0.4
Separator tortuosity	*τ* = 2.5

Initial lithium concentrations were set to reflect specific SOC values of 10%, 50%, and 90%, and were held constant during the simulations to emulate open-circuit storage conditions. Boundary conditions included zero-flux (Neumann) conditions at the electrode-current collector interfaces.

### Cell specification

The battery cell used in both the experimental study and simulation model was a commercial high-power cylindrical lithium iron phosphate/graphite (LiFePO_4_/C) cell with a nominal capacity of 2.5 Ah and a nominal voltage of 3.3 V. The cell employed LiFePO_4_ as the positive electrode (cathode) active material and graphite as the negative electrode (anode) active material. The key specifications of the tested cell are summarized in Table 3.

**Table 3:** Cell specifications

Item	Value
Type	Cylindrical
Dimensions	*Ø* 26 × 65 mm
Weight	76 g
Nominal capacity	2.5 Ah
Nominal voltage	3.3 V
Maximum voltage	3.6 V
Minimum voltage	2.0 V
Maximum-continuous charge current	10 A (4C)
Maximum-continuous discharge current	50 A (20C)
Storage temperature	−40 °C to 60 °C
Electrolyte	1 M LiPF_6_ in EC : EMC (3 : 7 by volume)
Anode	Graphite
Cathode	LiFePO_4_

### P2D model formulation

The electrochemical simulation of calendar aging was performed using the Pseudo-Two-Dimensional (P2D) model, a well-established framework for modeling lithium-ion batteries. This approach resolves the macroscopic (through-plane) and microscopic (particle-scale) transport phenomena that govern battery behavior under storage conditions. The model comprises a coupled set of partial differential equations (PDEs) that describe mass and charge conservation in both solid and liquid phases, as well as electrochemical reaction kinetics at the electrode/electrolyte interfaces.

The spatial domain was one-dimensional across the thickness of the cell (from anode current collector to cathode current collector), while each electrode domain included a spherical radial coordinate to simulate intra-particle diffusion. The general structure of the governing equations is described in Table 4.

**Table 4:** Governing equations and boundary conditions for P2D electrochemical model

Description	Equation	Boundary conditions	
Electrolyte phase transport	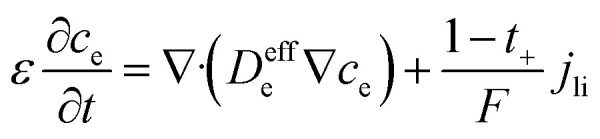	∇*c*_e_·*n*|_*x*=0,*L*_ = 0	(1)
Solid phase transport	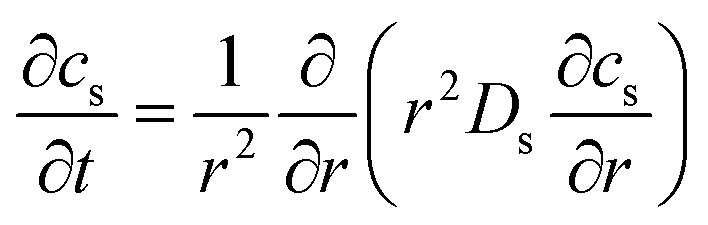	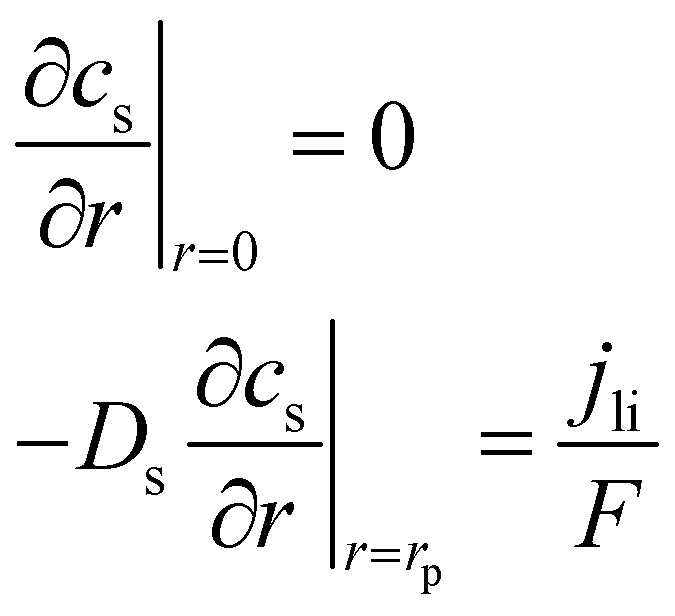	(2)
Charge conservation (electrolyte)	∇·(*κ*_eff_∇*ϕ*_e_) = −*j*_li_	∇*ϕ*_e_·*n*|_*x*=0,*L*_ = 0	(3)
Charge conservation (solid)	∇·(*σ*_eff_∇*ϕ*_s_) = *j*_li_	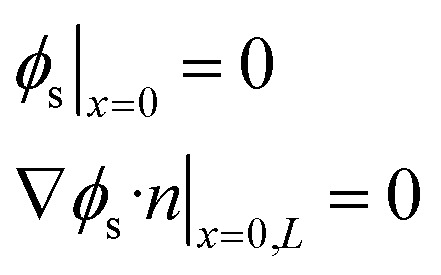	(4)
Electrode kinetics (Butler–Volmer equation)	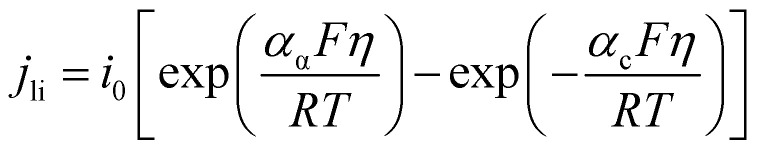		(5)
Reaction rate constant	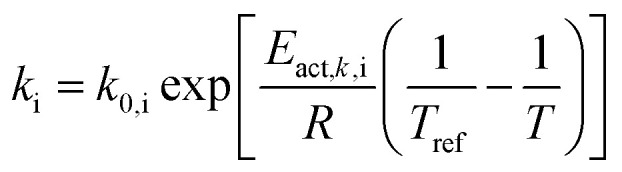 (ref. 34 and 35)	*E*_act,*k*,neg_ = 20 000 [J mol^−1^] (ref. 36)	(6)
		*E*_act,*k*,pos_ = 30 000 [J mol^−1^] (ref. 37)	
Liquid phase diffusion coefficient (diffusivity)	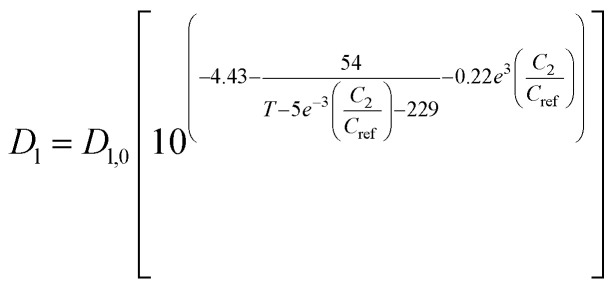	Similar to ref. 38 and 39	(7)
Solid phase diffusion coefficient	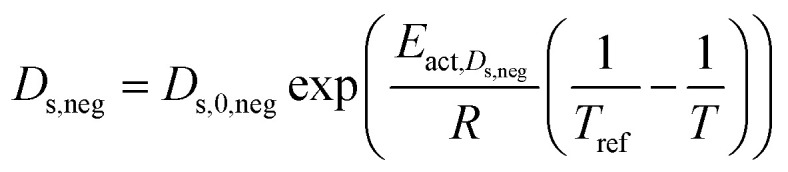 (ref. 35 and 37)	*E*_act,*D*_s_,neg_ = 68 025.7 [J mol^−1^]	(8)
	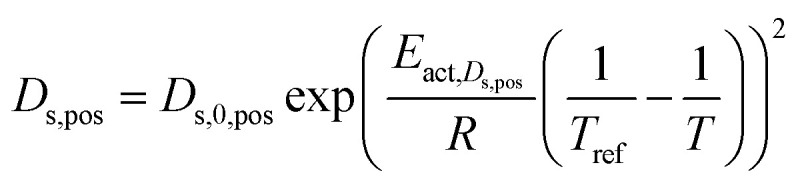 (ref. 38 and 39)	*E*_act,*D*_s_,pos_ = 3415 [J mol^−1^]	
		*E*_s,0,pos_ = 8.7 × 10^−14^ [m^2^ s^−1^]	
		*E*_s,0,neg_ = 1.52 × 10^−12^ [m^2^ s^−1^] (ref. 38 and 39)	

### Electrolyte conductivity loss model

One of the key degradation mechanisms during calendar aging of lithium-ion batteries is the reduction in ionic conductivity of the electrolyte. This loss in conductivity arises primarily from parasitic side reactions at the electrode/electrolyte interface, which lead to the formation of resistive byproducts—such as inorganic and organic species—that accumulate in the electrolyte and hinder lithium-ion mobility. As a result, ion transport through the porous media becomes progressively less efficient, contributing to capacity fade and increased internal resistance over time.^40,41^

In the P2D simulation framework, the time-dependent electrolyte conductivity *κ*(*t*) is modeled as a function of temperature *T*, state of charge (SOC), and storage duration *t*, incorporating both empirical aging observations and physical degradation trends. The degradation behavior follows a power-law dependence on time and an Arrhenius-type dependence on temperature, reflecting the thermally activated nature of the decomposition reactions. Additionally, a linear dependence on SOC is included to capture the role of higher electrode potentials in promoting side reactions.

The conductivity evolution is modeled by the following expression:9
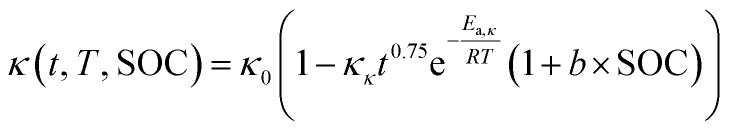
where *κ*(*t*, *T*, SOC) is effective ionic conductivity at time *t*, *κ*_0_ is initial conductivity of the fresh electrolyte (set to 10 mS cm^−1^), *κ*_*κ*_ = 1.5 × 10^−4^ s^−0.75^ empirical degradation rate constant, *n* = 0.75 is time exponent reflecting sub linear degradation behavior (self-inhibiting growth), and *b* = 0.015 is SOC sensitivity coefficient (dimensionless).^40^

This model captures the experimentally observed trends in which conductivity declines faster at elevated temperatures and higher SOC levels due to increased rates of solvent oxidation, salt decomposition, and gas evolution reactions.^42–44^ The exponent *n* = 0.75 is consistent with prior works that report non-linear, self-limiting degradation kinetics under storage conditions, particularly in systems dominated by SEI-related and electrolyte side reactions.^45^

By integrating this expression into the P2D model, the simulation accurately reflects the time-dependent transport limitations in the electrolyte, which in turn influence lithium-ion diffusion, overpotentials, and ultimately the rate of SEI growth and capacity fade.

### SEI thickness growth model

The growth of the Solid Electrolyte Interphase (SEI) layer on the surface of the graphite anode is a key contributor to capacity fade during calendar aging of lithium-ion batteries. The SEI is a passivation layer formed by the reductive decomposition of electrolyte components at low anode potentials, primarily during initial charging cycles and subsequently during long-term storage. Although this layer is essential for preventing continuous electrolyte degradation, it also consumes cyclable lithium and increases the interfacial resistance, both of which adversely affect battery performance.^46,47^

In the context of calendar aging, where the battery remains at open-circuit voltage and a fixed state of charge, SEI formation proceeds at a slow but steady rate due to persistent side reactions between the anode and the electrolyte. This process is thermally activated and strongly influenced by the state of charge (SOC), as higher SOCs correspond to lower anode potentials, which promote further reduction of electrolyte species.^48,49^

The SEI growth rate is linked to the side reaction current density *j*_SEI_, which is governed by a Tafel-type expression derived from Butler–Volmer kinetics:10
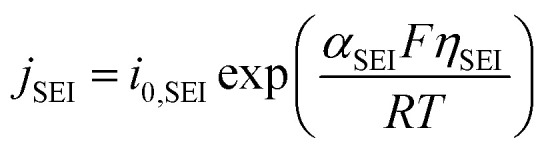
where *j*_SEI_ is the SEI formation current density, *i*_0,SEI_ = 1.0 × 10^−7^ A m^−2^ is the exchange current density, *α*_SEI_ = 0.5, and *η*_SEI_ = *ϕ*_s_ − *ϕ*_e_ − *U*_SEI_ is the SEI overpotential (*U*_SEI_ = 0.4 V).^49^

The cumulative growth of the SEI thickness over time is calculated from the integrated SEI formation charge:^40^11
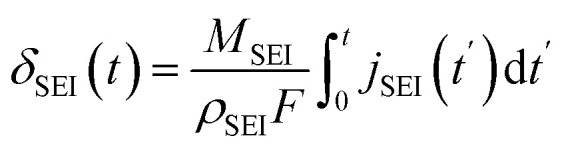
where *M*_SEI_ = 0.1 kg mol^−1^ is the molar mass of the SEI layer, *ρ*_SEI_ = 2000 kg m^−3^ is the SEI density. To capture the empirical observations that SEI growth slows down over time (*i.e.*, self-limiting behavior), the model adopts a power-law dependence with a time exponent of 0.75. This behavior is well-supported in the literature and reflects diffusion-limited growth of SEI components through the existing SEI layer.^40^ Furthermore, an Arrhenius term and a linear SOC dependence are incorporated to model thermal acceleration and potential-driven effects:12
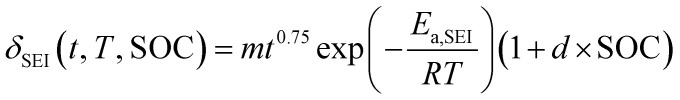
where *m* = 0.02 nm s^−0.75^, *E*_a,SEI_ = 55 kJ mol^−1^, and *d* = 0.012. The 0.75 exponent reflects the self-inhibiting nature of SEI growth.^47^

In this model, the SEI growth is directly linked to the loss of lithium inventory and hence to the capacity fade observed in long-term storage, as the lithium consumed in forming SEI is irreversibly lost from the electrochemically active pool. This coupling allows accurate prediction of capacity degradation as a function of time, temperature, and SOC within the P2D simulation environment.

### Model validation

The simulation study was performed across all five experimental stress condition combinations, encompassing three storage temperatures (40 °C, 47.5 °C, and 55 °C) and three state-of-charge (SOC) levels (10%, 50%, and 90%), and was further extended to include ambient temperature conditions at 25 °C for reference. Model validation was achieved by comparing the simulated capacity fade—derived from the loss of lithium inventory attributed to SEI layer formation—with experimental results reported in ref. 24. Capacity degradation was quantified using the relation:13
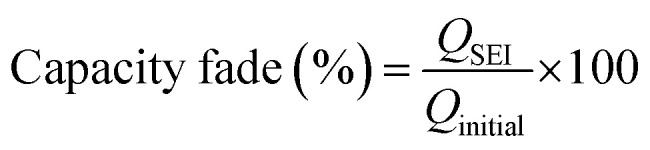
where 
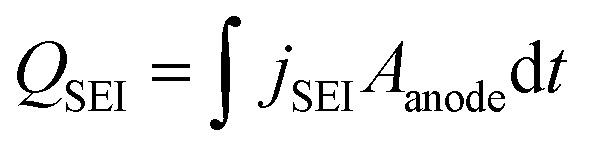
 represents the total charge consumed by SEI formation over time, and *Q*_initial_ = 2.5 Ah is the nominal initial capacity of the cell. The simulation results demonstrated strong agreement with the experimental data, and the root mean square error (RMSE) was employed as a quantitative metric to evaluate the accuracy of the model predictions.

**Fig. 1:**
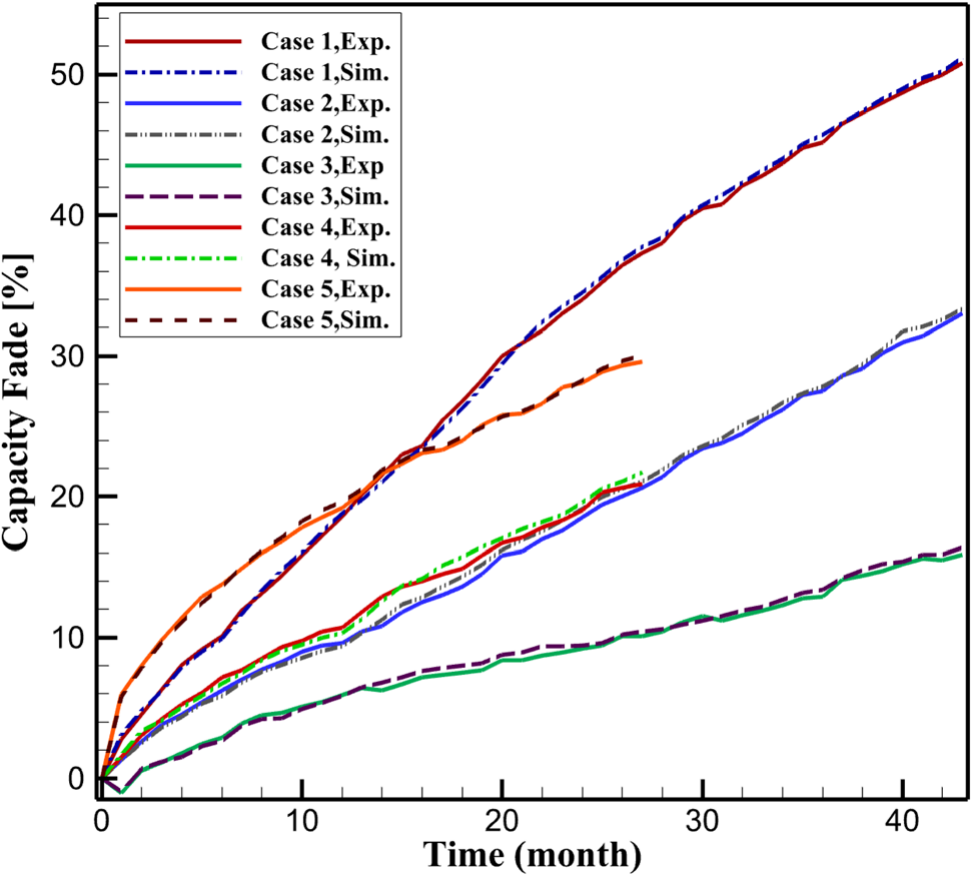
Comparison between the experimental and present study of capacity fade.

To elucidate the capacity fade model, the experimentally measured capacity during 1C-rate discharge tests, as reported in Sui *et al.*,^24^ is acknowledged to encompass both irreversible Loss of Lithium Inventory (LLI) and power fade resulting from increased internal resistance. Eqn (13) quantifies capacity fade predominantly through the charge consumed by SEI formation (QSEI), reflecting the primary LLI mechanism in calendar aging of LiFePO_4_/graphite cells. However, the P2D framework comprehensively accounts for kinetic limitations associated with power fade by dynamically simulating SEI thickness growth, which elevates interfacial resistance at the anode, and electrolyte conductivity loss, which increases transport resistance across the cell. These resistance-enhancing effects are fully integrated into the physics-based simulation, influencing lithium-ion transport and reaction kinetics. The robust agreement between simulated and experimental capacity fade, evidenced by RMSE values below 0.9 across all aging conditions (Fig. 1), validates the model's ability to capture the combined effects of LLI and power fade. By emphasizing LLI in eqn (13), the model provides a precise metric for the dominant irreversible degradation mechanism, while the P2D framework ensures a holistic representation of all pertinent degradation processes.

Fig. 1 compares simulated and experimental capacity fade for Cases 1–5, showing strong agreement with RMSE values of 0.8049 (Case 1), 0.8813 (Case 2), 0.3648 (Case 3), 0.4665 (Case 4), and 0.3413 (Case 5). Cases 1–3 (50% SOC at 40 °C, 47.5 °C, 55 °C) exhibit a clear trend of increasing capacity fade with temperature, consistent with Arrhenius-driven acceleration of SEI growth and electrolyte decomposition. Case 4 (10% SOC, 55 °C) shows the least fade, reflecting reduced side reaction rates at lower SOC. However, Case 5 (90% SOC, 55 °C) initially shows higher capacity fade than Case 1 (50% SOC, 40 °C) but falls below it after approximately 15 months. This behavior, observed in the experimental data, may result from the self-limiting nature of SEI growth at high SOC, where the thicker SEI layer (Fig. 3, 308.6 nm at 36 months) reduces further electrolyte reduction, slowing fade relative to temperature-driven degradation at 50% SOC. Additionally, experimental variations in cell preconditioning or SEI stabilization at high SOC could contribute to this trend.^24^ These results reinforce the nonlinear interplay of temperature and SOC, supporting the model's predictive capability.

**Fig. 2:**
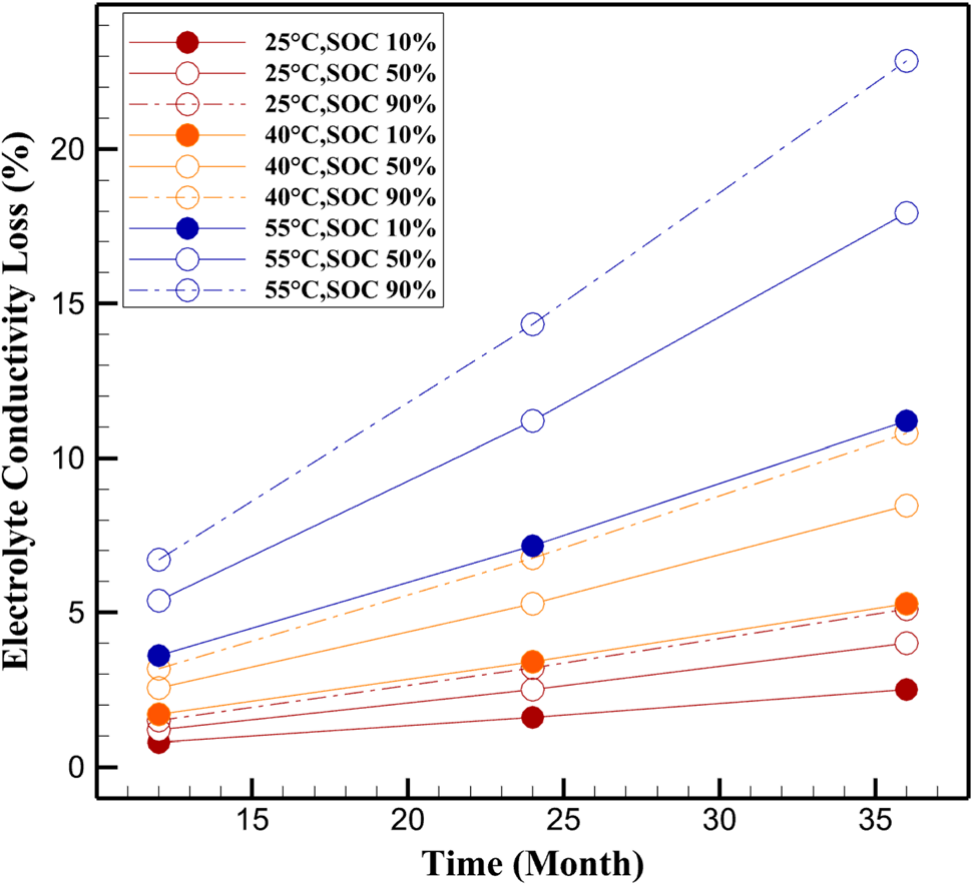
Electrolyte conductivity loss (%) for LiFePO_4_/C battery.

**Fig. 3:**
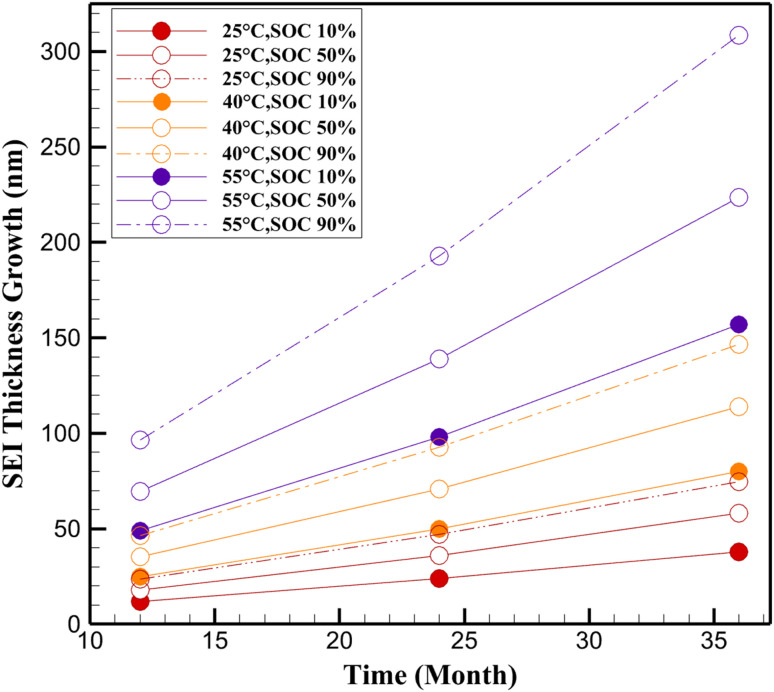
SEI thickness growth (nm) for LiFePO_4_/C battery.

**Fig. 4:**
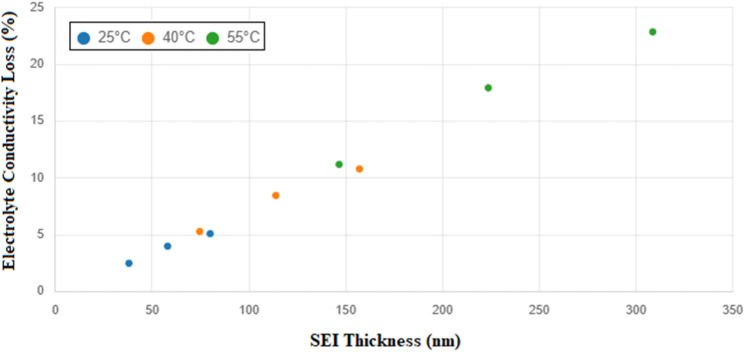
Interdependence of SEI thickness growth and electrolyte conductivity loss at 36 months.

**Fig. 5:**
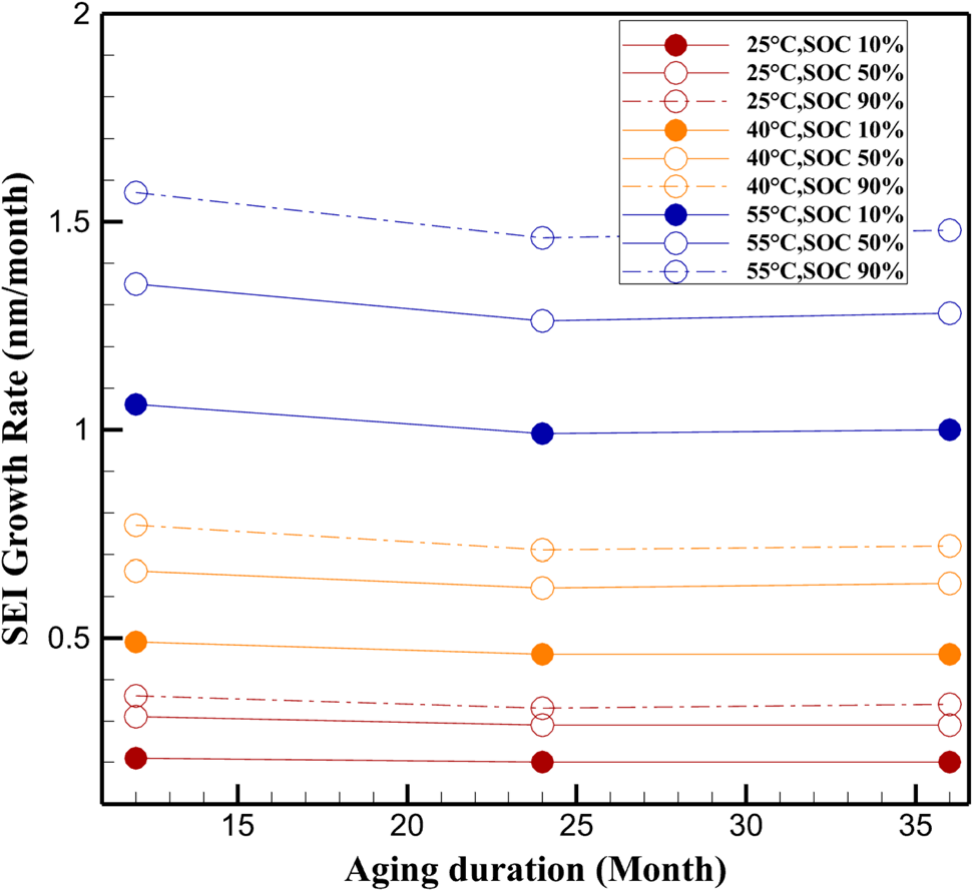
SEI growth rate (nm per month) at different temperatures and SOC levels.

The experimental data from Sui *et al.*^24^ utilized storage temperatures of 40 °C, 47.5 °C, and 55 °C to accelerate calendar aging, reflecting conditions relevant to high-stress applications. For model validation, simulations were conducted at these temperatures, achieving RMSE values below 0.9 (Fig. 1), confirming robust agreement with experimental capacity fade. Additionally, simulations at 25 °C were included as a reference condition to evaluate degradation under ambient storage, a scenario of practical interest for battery management but not covered in the experimental dataset.^24^ The 25 °C simulations extrapolate the validated model parameters (Tables 2–4) using Arrhenius and Tafel kinetics, providing predictive insights into low-temperature aging behavior, as shown in Fig. 2–5. This extension enhances the model's applicability across a broader range of storage conditions.

## Results and discussion

The validated Pseudo-Two-Dimensional (P2D) model was employed in COMSOL Multiphysics to simulate the progression of electrolyte conductivity loss and SEI layer growth in LiFePO_4_/C cells under various calendar aging conditions. Simulations were primarily conducted at 40 °C and 55 °C to align with the experimental conditions from Sui *et al.*,^24^ enabling direct validation against capacity fade data (Fig. 1). An additional simulation at 25 °C was included as a reference to assess aging under ambient storage conditions, complementing the experimental temperatures of 40 °C, 47.5 °C, and 55 °C. Results are presented for all three temperatures (25 °C, 40 °C, 55 °C) and SOC levels (10%, 50%, 90%) at 12, 24, and 36 months (Fig. 2–5). The model incorporates fundamental electrochemical processes, including SEI film formation and electrolyte decomposition, governed by temperature and SOC-dependent kinetics described by Arrhenius and Tafel formulations.

### Electrolyte conductivity loss

Electrolyte conductivity loss represents the reduction in ionic mobility within the electrolyte due to parasitic side reactions and electrolyte decomposition. These phenomena result in resistive by-products, which progressively impede lithium-ion transport and increase internal cell impedance. As shown in Fig. 2, the simulated results confirm the exponential sensitivity of conductivity loss to both temperature and SOC.

The pronounced increase in electrolyte conductivity loss at elevated temperatures is consistent with prior studies on thermally accelerated degradation in lithium-ion battery systems.^50^ As evidenced by the simulation results presented in Fig. 2, the rate of conductivity decline exhibits a clear exponential dependence on temperature. For instance, after 36 months of storage, conductivity loss at 55 °C reaches up to 22.86% at 90% SOC, compared to only 2.5% under ambient temperature (25 °C) and 10% SOC. This marked disparity highlights the critical role of thermal energy in promoting side reactions such as solvent decomposition, salt breakdown, and formation of resistive byproducts, which collectively hinder ionic transport through the electrolyte. In addition to temperature effects, the influence of state of charge (SOC) is shown to be equally significant. The data reveal that higher SOC levels (particularly above 50%) amplify conductivity loss across all temperature conditions. This behavior can be attributed to increased anode lithiation, which lowers the anode potential and accelerates the oxidative decomposition of the electrolyte.^51^ At 40 °C, for example, conductivity loss after 36 months increases from 5.29% at 10% SOC to 10.80% at 90% SOC, underscoring the compounding nature of SOC and temperature as degradation drivers.

The inclusion of 25 °C in simulations provides critical insights into calendar aging under ambient conditions, where degradation is significantly slower (*e.g.*, 2.5% conductivity loss and 38 nm SEI thickness at 10% SOC after 36 months, Fig. 2 and 3). These results, derived from the validated P2D model, suggest that storage at ambient temperatures can substantially extend battery lifespan, supporting optimized storage strategies for applications with prolonged idle periods.^24^ These results support the hypothesis that storage at elevated SOC and temperature synergistically exacerbates electrolyte decomposition and transport limitation mechanisms. The combined effects of thermally activated kinetics and high electrode potentials accelerate the accumulation of inert species within the electrolyte, thereby impeding lithium-ion mobility and contributing to long-term performance degradation. This underscores the importance of thermal and SOC management during storage to mitigate calendar aging and extend battery life.

### SEI thickness growth

The simulation results of SEI layer formation further elucidate the critical role of temperature and SOC in calendar aging of lithium-ion batteries. As shown in Fig. 3, SEI thickness exhibits a nonlinear growth pattern over time, with both temperature and SOC acting as accelerating factors. At 25 °C and 10% SOC, the SEI thickness increases moderately from 12.0 nm at 12 months to 38.0 nm at 36 months, reflecting a relatively stable degradation regime. However, under high-stress storage conditions (*e.g.*, 55 °C and 90% SOC), SEI thickness expands rapidly, reaching 308.6 nm after 36 months—more than an eightfold increase relative to the low-stress condition.

This exponential growth aligns with the diffusion-limited nature of SEI formation, where the rate of layer buildup is governed by both reaction kinetics and the gradual penetration of electrolyte decomposition products through the existing SEI. Elevated temperatures significantly enhance reaction rates at the anode/electrolyte interface, thereby intensifying SEI growth. Likewise, high SOC levels (particularly above 50%) correspond to lower anode potentials, which promote continuous electrolyte reduction and facilitate further SEI formation.

The compounding effect of temperature and SOC is evident when comparing mid- and high-SOC conditions. At 55 °C, SEI thickness at 50% SOC reaches 223.7 nm after 36 months, while increasing SOC to 90% accelerates SEI growth by over 38%, culminating in a 308.6 nm layer. These results support prior findings that elevated SOC enhances the availability of electrochemical driving forces for parasitic side reactions, thereby intensifying irreversible lithium consumption and capacity loss.

The SEI growth data substantiate the underlying model assumptions, including the adoption of a sub-linear (*t*^0.75^) time dependence and Arrhenius-type temperature scaling. This modeling framework effectively captures the self-limiting yet thermally activated nature of SEI formation, which is essential for predicting long-term lithium inventory loss under varied storage scenarios.

### Interdependence between SEI thickness growth and electrolyte conductivity loss

The degradation behaviors associated with SEI formation and electrolyte conductivity loss, while modeled as distinct processes, are intrinsically interlinked through shared underlying mechanisms rooted in electrode–electrolyte interfacial chemistry. Both phenomena are driven by parasitic side reactions that occur predominantly at the anode surface during storage, particularly under elevated temperature and SOC conditions. These side reactions consume electrolyte solvents and lithium ions, leading simultaneously to (i) the formation of the resistive SEI layer and (ii) the accumulation of non-conductive byproducts in the electrolyte, which impair ionic mobility.

The simulation results presented in Fig. 2 and 3 highlight a strong correlation between the rate of SEI growth and the magnitude of electrolyte conductivity loss. For instance, under the most aggressive aging condition (55 °C, 90% SOC), the SEI thickness increases to 308.6 nm after 36 months, while the corresponding conductivity loss reaches 22.86% (Fig. 4). In contrast, at 25 °C and 10% SOC, both metrics remain relatively low, with SEI thickness limited to 38.0 nm and conductivity loss to 2.5%. This trend indicates that as SEI formation accelerates, it not only depletes active lithium but also exacerbates electrolyte degradation, thereby reducing transport efficiency across the cell.

These findings suggest that SEI and electrolyte degradation are not independent phenomena but rather co-evolving and self-reinforcing processes. From a practical standpoint, this interdependence has significant implications for long-term battery storage strategies. Minimizing SEI formation through optimized SOC and thermal control not only preserves lithium inventory but also mitigates electrolyte transport degradation, thereby maintaining cell impedance within acceptable limits.

### Asymmetric behavior of SEI growth across SOC levels

The SEI growth behavior exhibits marked asymmetry across different SOC levels, a trend that becomes increasingly pronounced under elevated temperature conditions. As observed in Fig. 5, at 55 °C, the monthly SEI growth rate increases significantly with SOC. After 12 months, the growth rate at 90% SOC reaches 1.57 nm per month, which is approximately 48% higher than the corresponding rate at 10% SOC (1.06 nm per month). This asymmetry persists over longer storage periods, indicating a sustained acceleration of degradation under high SOC conditions.

This behavior can be attributed to the electrochemical potential of the anode, which decreases as SOC increases, thereby enhancing the thermodynamic driving force for electrolyte reduction reactions. As a result, a more aggressive SEI formation occurs, leading to faster lithium consumption and a thicker, more resistive interphase layer. Moreover, higher SOC levels typically correspond to a higher degree of lithiation in the graphite anode, where the electrolyte is more prone to reductive decomposition due to the lower interfacial potential.

The data from the current study (Fig. 5) reinforce the necessity of accounting for SOC-induced asymmetry in predictive battery aging models. For instance, the consistently higher SEI growth rates at 90% SOC across all time intervals suggest that time-averaged degradation estimates may significantly underestimate localized capacity loss in real-world scenarios, particularly for applications where batteries are regularly stored or operated at high charge levels.

## Conclusion

This study utilized a Pseudo-Two-Dimensional (P2D) electrochemical model to investigate calendar aging in LiFePO_4_/graphite lithium-ion batteries, focusing on SEI layer growth and electrolyte conductivity degradation. Simulation results reveal a nonlinear, synergistic impact of storage temperature and state-of-charge (SOC), with severe degradation at 55 °C and 90% SOC, where SEI thickness exceeded 300 nm and conductivity loss surpassed 20% after 36 months. The model, validated against experimental data with RMSE below 0.9, highlights the interdependence of SEI growth and electrolyte degradation, driven by parasitic side reactions. These findings underscore the importance of optimized storage conditions to mitigate aging and provide a scalable framework for predicting long-term battery performance, supporting enhanced battery design and management strategies.

## Conflicts of interest

There are no conflicts to declare.

## Data Availability

All data supporting the findings of this study, including experimental results, are derived solely from the data reported in Sui *et al.* (2021)^24^ and are included within the manuscript and its supplementary materials. No additional data beyond those presented in the article were required or used for this study.
